# Performance of the bispectral index and electroencephalograph derived parameters of anesthetic depth during emergence from xenon and sevoflurane anesthesia

**DOI:** 10.1007/s10877-022-00860-y

**Published:** 2022-04-19

**Authors:** Steven McGuigan, David A. Scott, Lisbeth Evered, Brendan Silbert, David T. J. Liley

**Affiliations:** 1 Department of Anesthesia and Acute Pain Medicine, St. Vincent’s Hospital Melbourne, 41 Victoria Parade, Melbourne, 3065 Australia; 2 Department of Anesthesiology, Weill Cornell Medicine, New York, USA; 3 Department of Critical Care, University of Melbourne, Melbourne, Australia; 4 Department of Medicine, University of Melbourne, Melbourne, Australia

**Keywords:** Xenon, Electroencephalogram, Sevoflurane, Depth of anesthesia monitoring, Frequency domain analysis, Bispectral analysis

## Abstract

Many processed EEG monitors (pEEG) are unreliable when non-GABAergic anesthetic agents are used. The primary aim of the study was to compare the response of the Bispectral Index (BIS) during emergence from anesthesia maintained by xenon and sevoflurane. To better understand the variation in response of pEEG to these agents, we also compared several EEG derived parameters relevant to pEEG monitoring during emergence. Twenty-four participants scheduled for lithotripsy were randomized to receive xenon or sevoflurane anesthesia. Participants were monitored with the BIS and had simultaneous raw EEG collected. BIS index values were compared at three key emergence timepoints: first response, eyes open and removal of airway. Two sets of EEG derived parameters, three related to the BIS: relative beta ratio, SynchFastSlow and SynchFastSlow biocoherence, and two unrelated to the BIS: spectral edge frequency and the composite cortical state, were calculated for comparison. BIS index values were significantly lower in the xenon group than the sevoflurane group at each emergence timepoint. The relative beta ratio parameter increased significantly during emergence in the sevoflurane group but not in the xenon group. The spectral edge frequency and composite cortical state parameters increased significantly in both groups during emergence. The BIS index is lower at equivalent stages of behavioural response during emergence from xenon anesthesia when compared to sevoflurane anesthesia, most likely due to differences in how these two agents influence the relative beta ratio. The spectral edge frequency and composite cortical state might better reflect emergence from xenon anaesthesia.

*Clinical trial number and registry* Australia New Zealand Clinical Trials Registry Number: ACTRN12618000916246.

**Supplementary Information:**

The online version contains supplementary material available at 10.1007/s10877-022-00860-y.

## Introduction

Although originally conceived as monitors to reduce the incidence of awareness, processed electroencephalogram (pEEG) devices are increasingly being utilized as tools to guide anesthesia delivery to achieve what is hypothesized to be an ideal depth of hypnosis [1, 2].

One such monitor, the Bispectral Index monitor (BIS) (Medtronic, Minneapolis, USA), utilizes time and frequency domain parameters as well as the bispectrum, a measure of inter-frequency phase synchrony, to produce a single index value [3]. The parameters that define this index were selected for their ability to statistically discriminate between different behavioral states and are reasonably well described in the literature [4, 5]. The BIS index has been validated in volunteer studies utilizing agents that act predominantly at the γ-aminobutyric acid–mediated (GABAergic) receptor such as propofol, isoflurane and midazolam [6].

Despite the development of a wide range of pEEG monitors, and multiple updates to monitors such as the BIS, pEEG monitors remain significantly less reliable when anesthetic agents with predominantly non-GABAergic molecular targets are used [7]. For example, the BIS value is not reduced from baseline following the administration of a ketamine induction dose [8]. Both state and response entropy values (GE Healthcare, Helsinki, Finland) display high inter- and intra-individual variability during ketamine anesthesia, rendering the entropy monitor unreliable [9]. Ketamine given in conjunction with GABAergic anesthesia can also cause a paradoxical increase in the BIS index, and both state and response entropy [10, 11]. Administration of nitrous oxide, alone or in conjunction with other agents, has little effect on the BIS index [8, 12]. There is also insufficient data to validate the use of other pEEG monitors during non-GABAergic anesthesia [13].

The failure of pEEG monitors to respond uniformly to the administration of different anesthetic agents likely reflects the diverse changes in the EEG induced by anesthetic agents which modulate different molecular targets [14]. For example, ketamine and nitrous oxide increase higher frequency beta and gamma activity in the EEG whilst surgical depth of anesthesia with GABAergic agents is associated with reduced higher frequency activity [14, 15]. Even amongst agents that are understood to act primarily on the same receptor, such as the halogenated volatiles and propofol, there can be significant variation in EEG spectral features [16].

The noble gas xenon is the only inhalational agent capable of providing reliable surgical depth of anesthesia in normobaric conditions that has a predominantly non-GABAergic action [17]. During maintenance anaesthesia, reductions in the BIS appear to be similar for xenon and the volatile agents isoflurane and sevoflurane [18–21]. However, significantly lower BIS values have been reported at equivalent behavioral stages during emergence from xenon anesthesia when compared to volatile anesthesia [18, 20, 21].

We have previously demonstrated that maintenance anesthesia with xenon and sevoflurane are associated with distinct EEG spectral features [22]. It remains to be seen if these distinct spectral features persist into the emergence phase, and if so, whether these differences in spectral features influence the EEG parameters from which the BIS is derived. There is also a need to identify alternative EEG derived parameters that might better predict responsiveness during emergence from agents with predominantly GABA mediated and non-GABAergic mediated actions. The emergence phase offers an opportunity to study the EEG characteristics associated with reductions in anesthetic depth. Prompt recognition of these changes is essential to the prevention of awareness throughout the anesthetic episode.

The aim of this study is to evaluate the response of the BIS index, and the parameters that inform this index, during emergence from two anesthetics with different molecular targets, xenon and sevoflurane, following a standard clinical procedure. The primary hypothesis is that BIS values will be lower in the xenon group at equivalent stages of responsiveness during emergence. The secondary hypothesis is that EEG parameters during emergence that inform the BIS will differ between the groups.

## Methods

This study was designed as a single center, single-blinded, randomized control trial with several prospective outcomes that included neural biomarkers as well as raw and processed EEG. A comparison of the maintenance EEG and perioperative neural biomarkers of the treatment groups have previously been published [22, 23]. The study was approved by the Hospital’s Institutional Review Board (Approval number HREC/18/SVHM/221) and written informed consent was obtained from all subjects participating in the trial. The trial was registered prior to patient enrollment at ANZCTR.org.au (ACTRN12618000916246).

Twenty-four patients aged 50 years and over and scheduled for extra-corporeal shock wave lithotripsy (ESWL) of renal stones were enrolled for the trial. Exclusion criteria were a diagnosed neurocognitive condition, high risk of postoperative nausea and vomiting (PONV), contraindication to the use of a laryngeal mask airway, severe respiratory disease (unable to tolerate inspired oxygen concentration of 35%) or any contraindication to anesthesia with a volatile agent.

A Bispectral Index (BIS) electrode was placed on the forehead of each participant prior to the initiation of anesthesia. Electrodes were connected via the BIS LoC 2 Channel OEM Module (Medtronic, Minneapolis, USA) and values displayed on a Philips Intellivue MX800 monitor (Philips North America, Massachusetts, USA). A signal quality index of greater than 90% was confirmed prior to the induction of anesthesia. Each participant also had a second electrode montage placed on the contralateral forehead for recording of the raw EEG (Brain Anesthesia Response Monitor (BAR), Cortical Dynamics, Perth). The BAR electrodes were located at approximately Fpz and Fp1/Fp2 according to the extended 10–20 EEG electrode placement system [24].

BIS values were recorded at 1-min intervals for the entire anesthetic episode, from 5 min prior to induction of anesthesia until participants were ready for transfer to the post-anesthesia care unit. The BIS index value of each participant was also recorded at the following predetermined key stages of the procedure: induction, commence inhalational agent, maintenance (calculated as the mean of all values from commence inhalational until cease inhalational), cease inhalational agent, first response to voice, opening of eyes, removal of laryngeal mask airway. Measurements in which signal quality was lower than 90% or EMG was over 30% were discarded to ensure fidelity of the recordings.

Medical grade xenon was provided by Coregas Australia (Coregas, Thomastown, Australia). Xenon anesthesia was administered using the Akzent X Color (Stephan, Gackenbach, Germany) anesthetic machine in a semi-closed circle circuit. Xenon was approved for use as an inhalational anesthetic agent by the European Medicines Evaluation Agency in March 2007. A Clinical Trial Notification for its use in this study was approved by the Australian Therapeutic Goods Administration. Sevoflurane anesthesia was administered using the Draeger Primus (Draeger Australia Pty. Ltd., Victoria) in a low-flow circle circuit.

All participants received preoxygenation via a circle circuit for 5 min prior to induction. A remifentanil infusion at a dose of 0.1 mcg kg^−1^ min^−1^ was commenced simultaneously with preoxygenation. Anesthesia was induced with a bolus of propofol 2 mg kg^−1^. Following induction, an appropriately sized laryngeal mask airway was placed. Additional boluses of propofol could be given at the discretion of the anesthesiologist to facilitate airway placement.

Anesthesia was maintained with a target end-tidal concentration of 60% xenon in the xenon group and a target end-tidal concentration of 0.9 of the age adjusted MAC of sevoflurane in the sevoflurane group. An end-tidal concentration of 60% xenon represents a MAC of approximately 0.9 [25]. There are no validated age-adjusted MAC values for xenon [25]. The inspired oxygen concentration was 35%, achieved by blending with air for both groups. Participants’ lungs were ventilated with a tidal volume of 6 ml kg^−1^ and the respiratory rate titrated to achieve normocapnea.

In the event of hemodynamic changes suggestive of inadequate or excessive anesthesia (change in MAP or HR > 20% from baseline) the remifentanil infusion rate was titrated accordingly. Titration of the remifentanil rate and additional propofol boluses could also be administered at the discretion of the anesthesiologist in response to evidence of inadequate anesthetic depth (e.g. movement, coughing or laryngospasm). Participants received acetominophen 1 g IV intraoperatively for postoperative analgesia and droperidol 625 mcg and ondansetron 4 mg at the conclusion of the procedure for anti-emetic prophylaxis.

Following the conclusion of the ESWL procedure the inhalational agent and remifentanil were ceased. At this time a blinded member of the research staff spoke the words ‘*[first name]*, open your eyes’ to the participant. This phrase was repeated at 30 s intervals throughout the emergence phase until the laryngeal mask airway was removed. A second blinded member of the research staff recorded the time and BIS values at the following key points of emergence, participants’ first response, participants’ eyes open and removal of the laryngeal mask. The laryngeal mask was removed at the discretion of the anesthesiologist in accordance with our normal practice (i.e. patient spontaneously ventilating, responding to voice and opening eyes). Following this, participants were transferred to the post-anesthetic care unit for routine postoperative monitoring.

Following discharge, participants were contacted by telephone by a member of the research team and a modified Brice questionnaire to identify any possible awareness was administered.

## EEG parameter analysis

Raw EEG recordings at a sampling frequency of 480 Hertz (Hz) were obtained from the BAR monitor using data communication protocols provided by the manufacturer. The raw EEG was bandpass filtered at 0.5–50 Hz. EEG analysis was performed in MATLAB 2020a (The Mathworks, Natick, MA) using custom scripts and the Higher Order Spectral Analysis [26] and Chronux [27] toolboxes. Spectral power was calculated using the multitaper method (time-bandwidth product = 3, number of tapers = 5) on contiguous 2-s segments with no overlap. Group median spectrograms for emergence were calculated by taking the median value of the power in each frequency bin, as computed using the multitaper method described above, across all participants in each group. Linear interpolation was utilised to normalise the multitaper spectrum of each participant for emergence time. The power in the frequency bins corresponding to four frequency bands, delta (0.5–4 Hz), theta (4–8 Hz), alpha (8–12 Hz) and beta (12–30 Hz), was summed for each participant and the median value in each group taken to plot changes in band power over the emergence period.

Two groups of EEG parameters related to pEEG monitoring were calculated. The first group were those reported to be parameters that inform the BIS index (the relative beta ratio (RBR), SynchFastSlow (SFS) and SynchFastSlow biocoherence (SFS-C) [5]) and the second group represent parameters not involved in calculation of the BIS but which have been demonstrated to quantify anesthetic agent effect [spectral edge frequency 95 (SEF95) and composite cortical state (CCS)].

For the first group, the RBR was calculated as the logarithm of the ratio of spectral power between 30 and 47 Hz and the spectral power between 11 and 20 Hz [4, 5].

SFS was calculated as the logarithm of the ratio of the bispectrum between 40 and 47 Hz and the bispectrum between 0.5 and 47 Hz [4, 5]. Because the calculation of the bispectrum conflates both signal power and inter-frequency phase coupling [28] we also calculated the SynchFastSlow bicoherence. SFS-C was calculated as the logarithm of the ratio of the bicoherence between 40 and 47 Hz and the bicoherence between 0.5 and 47 Hz [28]. A non-zero bicoherence value between a pair of frequencies implies that they have a common phase and is considered a more specific measure of non-linear changes in electroencephalographic dynamics [3]. Statistically meaningful values of the bispectrum and the bicoherence were calculated by averaging over eight 2-s segments with 50% overlap.

The second group of EEG parameters represent those not involved in calculation of the BIS. The SEF95 has been utilized as a measure of depth of anesthesia but lacks the sensitivity and specificity to accurately predict responsiveness during anesthesia as a stand-alone measure. Despite this, the SEF95 correlates well with the BIS index [5] and is displayed by another commonly utilized pEEG monitor, the SEDline (Masimo Corporation, Irvine CA, USA) [29]. The SEF95 was calculated as the frequency below which 95% of the total spectral power between 0.5 and 47 Hz was found. The CCS measure is based on a neurophysiological model of cortical activity and has been proposed as an alternative to statistically derived indices such as the BIS [30]. One proposed benefit of this neurophysiological derived approach is accurate monitoring of a wider range of agents, including NMDA antagonists [31]. The unscaled composite cortical state (CCS) was calculated from the raw EEG using custom MATLAB code based on its description in Kuhlmann and Liley [31].

### Statistical analysis

All statistical analysis was performed using Stata (Stata/IC 16.0, StataCorp, Texas, USA). Parametric data were tested with the Student’s t-test and are presented as the mean and standard deviation. Non-parametric tests were utilized for the analysis of BIS values and EEG parameter values due to non-normality. BIS index values are presented as the median and interquartile range. Comparison of BIS values and EEG parameter values between anesthetic groups was performed with a Wilcoxon rank sum test. Comparison of BIS values and EEG parameter values within anesthetic groups was performed with a Wilcoxon sign rank test. The time course for emergence in each case was normalized to enable effective comparisons. Standard error in the median was bootstrap estimated using 5000 replicates. A *P* value < 0.05 was taken to indicate statistical significance.

The sample size of the study was determined for a related study measuring biomarkers in the same participants. No a priori sample size was performed for this exploratory analysis.

## Results

**Fig. 1:**
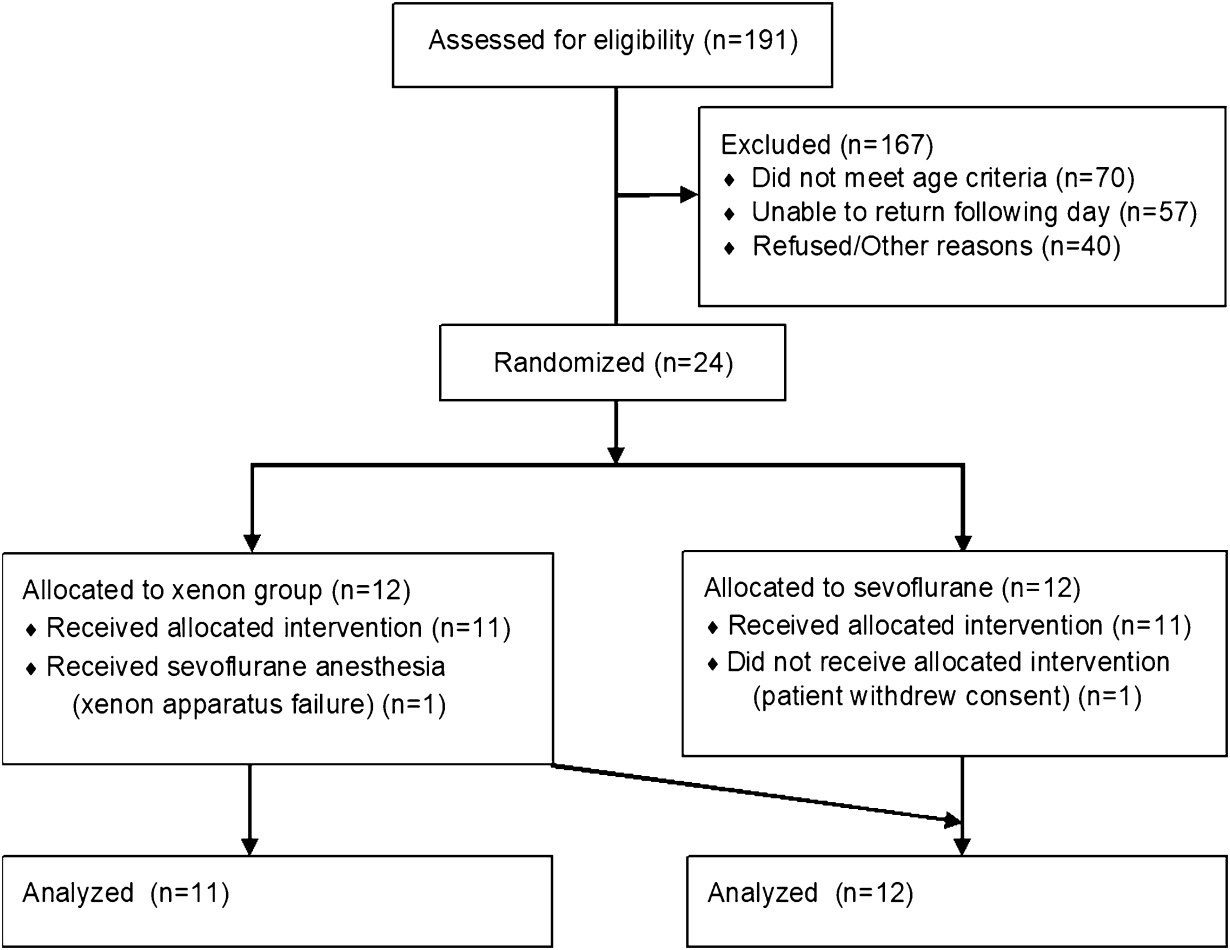
CONSORT diagram

A total of 24 participants were recruited for the study, 12 in each group. One participant randomized to the sevoflurane group withdrew consent prior to the administration of the anesthetic. One participant allocated to the xenon group received sevoflurane due to a malfunction of the xenon delivery apparatus identified prior to the procedure. The results below are based on a per-protocol analysis (Fig. 1).

## Patient and anesthesia characteristics

There was no significant difference identified between participant groups with respect to age, height, weight, sex and ASA status (Table 1). No statistically significant difference was identified in the duration of anesthetic, total propofol or total remifentanil dose (Table 1). The mean time in minutes from cessation of inhalational agent to opening eyes to voice (emergence) was significantly shorter in the xenon group when compared to the sevoflurane group, 5.4 (0.6) minutes (mean (SD)) versus 11.0 (1.8) min (*P* < 0.01).

**Table 1:** Patient and anesthesia characteristics for xenon and sevoflurane groups

	Xenon N = 11	Sevoflurane N = 12	*P* value
Age (year)	60 (6.3)	62 (9.1)	0.56
Height (cm)	170 (7.0)	170 (9.6)	0.92
Weight (kg)	77 (16.0)	78 (11.8)	0.85
Sex; male	6 (55%)	10 (83%)	0.17
ASA; I	2 (18%)	1 (8%)	0.59
Total propofol (mg)	257.3 (74.6)	215.8 (52.5)	0.14
Total remifentanil (µg)	513.9 (318.8)	479.4 (286.3)	0.79
Duration anaesthetic (minutes)	44.5 (15.8)	44.6 (5.3)	0.98
End tidal xenon (%)	56.6 (2.6)		
End tidal sevoflurane (%)		1.72 (0.2)	
Recovery (minutes)	5.4 (2.0)	11.0 (5.3)	< 0.01

Data are presented as mean (SD) or n (%)

## Comparison of BIS values between treatment groups

**Fig. 2:**
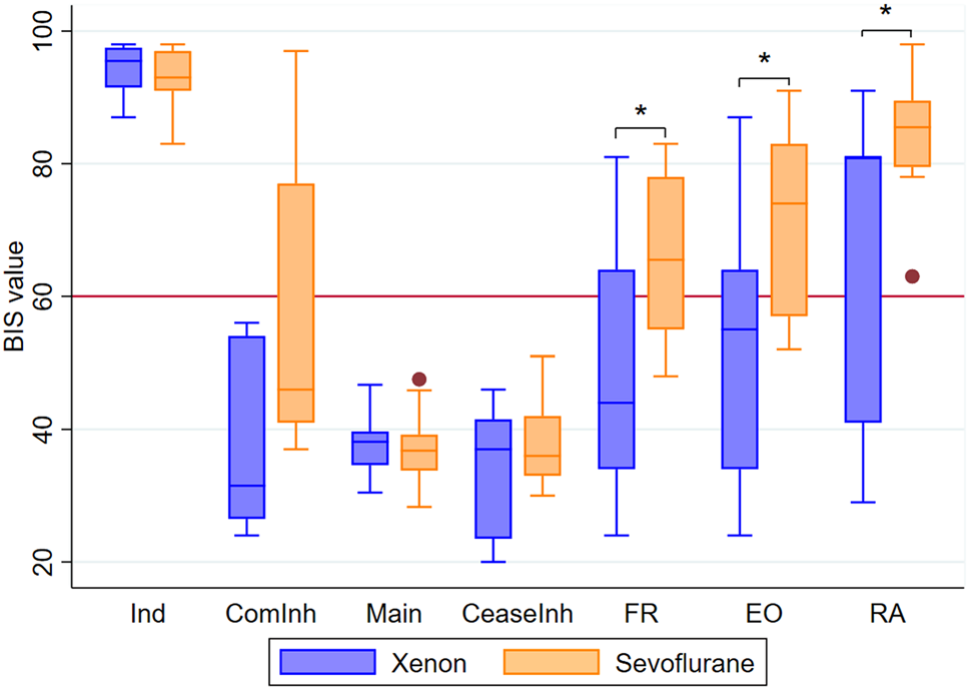
Comparison of BIS values between xenon (blue) and sevoflurane (orange) groups during anesthesia and emergence. Median represented by line within box and the bounds of the boxes represent the 25th and 75th centiles. The whiskers represent values within 1.5 × the interquartile range and outside values (represented by dots) are values beyond this. Statistically significant differences (Wilcoxon rank sum) are indicated by an asterisk (*P = 0.03). *Ind*  induction, *ComInh*  commence inhalational, *Main* maintenance, *CeaseInh* cease inhalational, *FR* first response, *EO* eyes open, *RA* removal of airway, *BIS* Bispectral index

The BIS values at all three emergence timepoints were significantly lower in the xenon group when compared to the sevoflurane group (Fig. 2): first response to voice, median BIS 44.0 (IQR 34.0–64.0) versus 65.5 (IQR 55.0–78.0) (*P* = 0.03), eyes open, median BIS 55.0 (IQR 34.0–64.0) versus 74.0 (IQR 57.0–83.0) (*P* = 0.03), and removal of airway, median BIS 81.0 (IQR 41.0–81.0) versus 85.5 (IQR 80.0–90.0) (*P* = 0.03).

There was no statistically significant difference between BIS values of the xenon and sevoflurane groups at the induction, commence inhalational or cease inhalational time points. There was also no significant difference in the mean value of the BIS for the maintenance period between groups (Fig. 2).

## Comparison of power spectral density between treatment groups

**Fig. 3:**
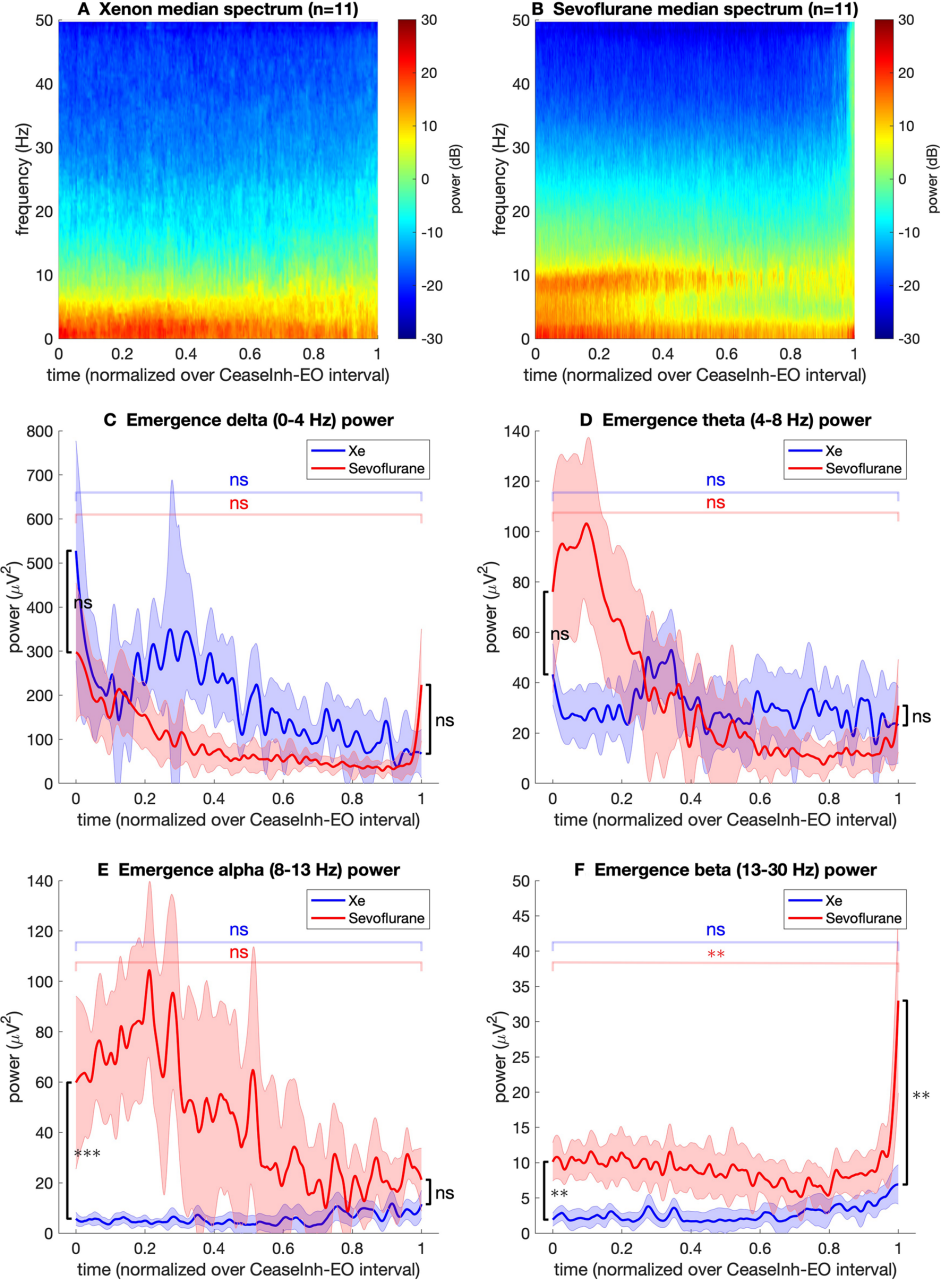
Group median spectrograms and median power corresponding to delta, theta, alpha and beta frequency bands during emergence in the xenon (blue) and sevoflurane (red) groups. Shaded areas indicate the bootstrap estimated (number of replicates = 5000) standard error of the median. The emergence time course for each participant was normalised on a scale from 0 (cease inhalational) to 1 (eyes open). Comparison of median frequency band power between anesthetic groups (Wilcoxon rank sum) at cease inhalational and eyes open indicated in black. Comparison of median frequency band power value at cease inhalational and eyes open (Wilcoxon sign rank) within xenon group (blue) and within sevoflurane group (red) indicated. *ns*  non-significant, *P < 0.05, **P < 0.01, ***P < 0.001

The group median spectrograms for the emergence phase in both groups are presented in Fig. 3(panels A and B). One participant in the sevoflurane group was excluded from power spectral density analysis due to significant raw EEG artifact. Therefore, power spectral density analysis was performed for 11 participants in each group. In the xenon group, spectral power over 20 dB was limited to the delta and theta bands (0.5–8 Hz). In the sevoflurane group, spectral power over 20 dB was observed in the delta, theta (4–8 Hz) and alpha (8–12 Hz) band.

The median value for power corresponding to the delta, theta, alpha and beta frequency bands over the emergence period for each group is presented in Fig. 3 panels C–F. The emergence time, from cease inhalational agent to eyes open, was different for each participant. Therefore, to compare the emergence trajectories of participants the emergence time period for each participant was normalised and is presented as a ratio from time 0 (cease inhalational), to time 1 (open eyes).

Alpha and beta power were significantly greater in the sevoflurane group when compared to the xenon group at cease of inhalational agent. At eye opening there was significantly greater beta power in the sevoflurane group when compared to the xenon group. There was significantly greater beta power at eyes open in the sevoflurane group when compared to cease inhalational.

## Comparison of EEG parameters during emergence between treatment groups

**Fig. 4:**
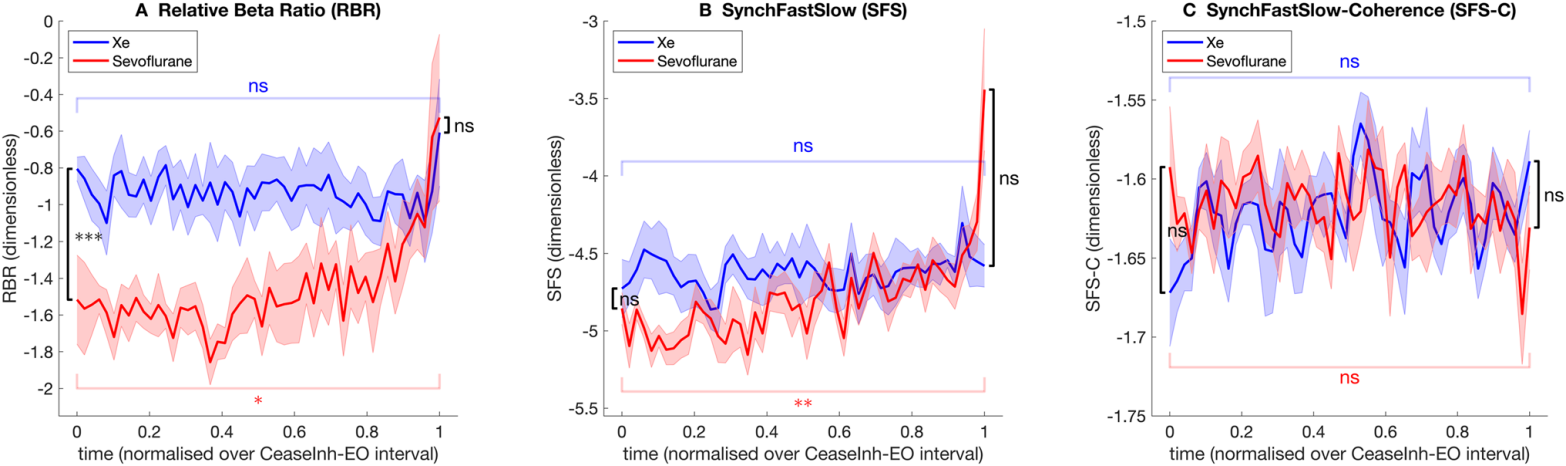
Trajectory of group median BIS-related parameter values during emergence from xenon (blue) and sevoflurane (red) anesthesia. Shaded areas indicate the bootstrap estimated (number of replicates = 5000) standard error of the median. The emergence time course for each participant was normalised on a scale from 0 (cease inhalational) to 1 (eyes open). Comparison of median parameter value between anesthetic groups (Wilcoxon rank sum) at cease inhalational and eyes open indicated in black. Comparison of median parameter value at cease inhalational and eyes open (Wilcoxon sign rank) within xenon group (blue) and within sevoflurane group (red) indicated. ns = non-significant, *P < 0.05, **P < 0.01, ***P < 0.001

**Fig. 5:**
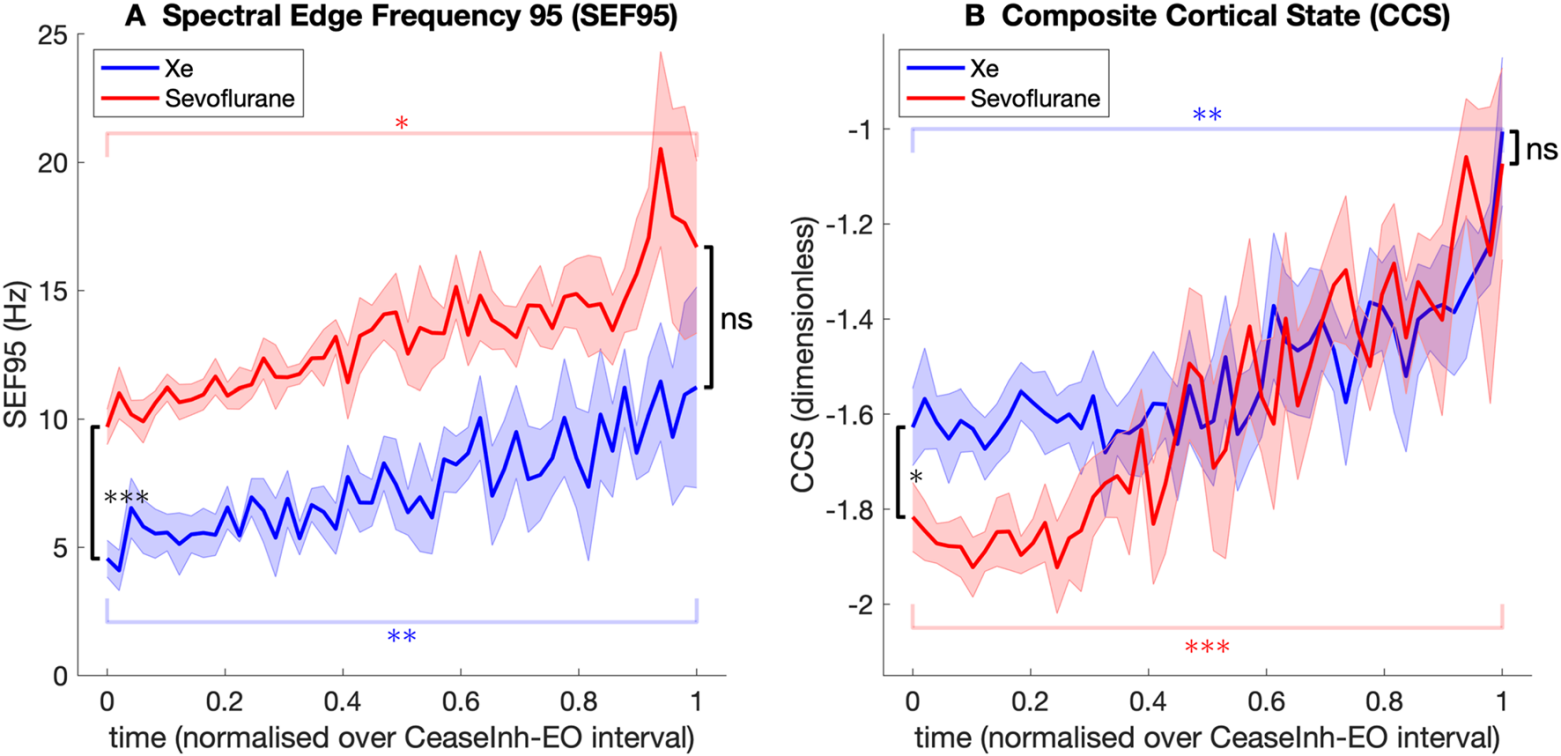
Trajectory of group median non-BIS related parameter values during emergence from xenon (blue) and sevoflurane (red) anesthesia. Shaded areas indicate the bootstrap estimated (number of replicates = 5000) standard error of the median. The emergence time course for each participant was normalised on a scale from 0 (cease inhalational) to 1 (eyes open). Comparison of median value between anesthetic groups (Wilcoxon rank sum) at cease inhalational and eyes open indicated in black. Comparison of median parameter value at cease inhalational and eyes open (Wilcoxon sign rank) within xenon group (blue) and within sevoflurane group (red) indicated. *ns* non-significant, *P < 0.05, **P < 0.01, ***P < 0.001

The median trajectory of participants for each of the calculated EEG parameters during emergence for the two treatment groups are presented in Figs. 4 and 5.

### BIS-related EEG parameters

The relative beta ratio (RBR) was significantly lower in the sevoflurane group compared to the xenon group at cease inhalational (Fig. 4A). During the second half of the emergence, from emergence time 0.5 to 1, the RBR increased in the sevoflurane group and the value at eyes open was significantly greater than at cease inhalational (*P* = 0.014). In contrast, there was no statistically significant difference in RBR values in the xenon group at cease inhalational and eye opening (*P* = 0.413) (Fig. 4A).

In the sevoflurane group the SynchFastSlow (SFS) was significantly greater at eye opening than at cease inhalational (*P* = 0.002), although this change appeared to immediately precede eye opening. There was no significant difference in the SFS values between cease inhalational and eye opening in the xenon group (Fig. 4B).

There was no statistically significant change in SynchFastSlow biocoherence (SFS-C) between cease inhalational and eye opening in either group (Fig. 4C).

### Non-BIS related EEG parameters

The spectral edge frequency (SEF95) increased over the emergence period in both anesthesia groups and the values at eyes open for both were significantly greater than at cease inhalational (xenon, *P* = 0.010, sevoflurane, *P* = 0.024) (Fig. 5A).

The composite cortical state (CCS) was significantly greater at eye opening than at cease inhalational in both groups (xenon, *P* = 0.010, sevoflurane, *P* = 0.001). The trajectory of both groups appears similar with the CCS relatively unchanged for the first quarter of the emergence phase, emergence time 0–0.25, before increasing steadily prior to eye opening (Fig. 5B).

## Brice questionnaire

Two participants in the sevoflurane group could not be contacted to administer the questionnaire. All remaining participants completed the questionnaire. No participants in the xenon or sevoflurane groups reported either memories or dreams between induction and emergence from anesthesia.

## Discussion

Despite the introduction of a wide range of pEEG monitors and their promotion as tools for preventing awareness and guiding anesthesia delivery, monitoring of anesthetic depth when non-GABAergic agents are utilised remains a significant challenge for pEEG technology. To evaluate the performance of the BIS during non-GABAergic anesthesia we utilised xenon gas, the only inhalational non-GABAergic agent capable of providing surgical depth of anesthesia. We confirmed previous findings that BIS index values were significantly lower at equivalent stages of responsiveness during emergence from xenon anesthesia when compared to sevoflurane anesthesia. We identified that the trajectory of the EEG parameter most relevant to the BIS index during emergence, the relative beta ratio, was significantly different in the two groups. We also identified alternative EEG parameters which may better reflect emergence from non-GABAergic agents.

Reliable identification that a patient receiving anesthesia has transitioned from a state in which they are not responsive to external stimuli to a state in which they are responsive to external stimuli is essential for any device designed to reduce the incidence of awareness or guide anesthesia delivery. Our findings suggest that patients can transition from a state of non-responsiveness to a state of responsiveness during xenon anesthesia with BIS values within the recommended range for surgical anesthesia. The BIS values at which participants transitioned to responsiveness were significantly lower in the xenon group when compared to the sevoflurane group.

There have been contradictory results in those studies that have compared the values of the BIS during emergence from xenon and sevoflurane anesthesia, with one study finding no statistically significant difference [19] and two others showing significantly lower values in the xenon group [20, 21]. All three studies utilised the neuromuscular blocking agent rocuronium.

The effects of neuromuscular blockade on the BIS index are controversial and a volunteer study designed to validate the BIS index was performed in the absence of muscle relaxants [6]. In a novel study of the effect of muscle relaxants on the BIS index, anaesthesiologist volunteers were administered muscle relaxant in the absence of a hypnotic agent. Despite participants maintaining a response to voice, confirmed by the isolated forearm technique, the administration of muscle relaxant was associated with BIS index values within the recommended range for surgical anesthetic depth [32]. Reversal of muscle relaxants might also effect changes in the BIS index. Reversal of atracurium with neostigmine has been associated with increased BIS values at a steady state of anesthesia [33]. In contrast, reversal of rocuronium with sugammadex at a steady state of anesthesia was not associated with any change in BIS values [34]. The current study benefited from the omission of neuromuscular blocking agents, and their reversal counterparts, eliminating neuromuscular blockade as a potential confounder.

To better understand the phenomenon of lower BIS values during xenon anesthesia emergence we performed power spectral density analysis. The group median spectrograms confirmed that distinct spectral features of xenon maintenance anesthesia which have previously been described, such as reduced alpha power when compared to sevoflurane anesthesia, [22] persist into the emergence phase.

To objectively investigate how these spectral differences during emergence might influence pEEG monitoring we calculated several EEG parameters utilized to assess anesthetic depth. Two of these EEG parameters are reported to contribute directly to the BIS index [5]. The relative beta ratio (RBR) is the parameter most strongly correlated with the BIS during light sedation [4]. Whilst the RBR increased steadily during the second half of the emergence phase in the sevoflurane group, there was no significant change in this parameter during the emergence phase in the xenon group. The different trajectories of the RBR during emergence likely contributes to the discrepancy in BIS values during emergence from these anesthetic agents.

The SynchFastSlow (SFS) is the sub-parameter that correlates most closely with the BIS index in the recommended range for general anesthesia [5]. We found that this parameter remained relatively unchanged during the emergence period for both anesthetic agents, although it did increase immediately prior to eye opening in the sevoflurane group.

The SynchFastSlow coherence (SFS-C), although not a parameter which directly contributes to the BIS index, [5] arguably represents a more accurate measure of phase synchrony than the SFS [4, 28]. The SFS-C applies a correction for the amplitude of the frequency components for which phase synchrony is quantified [4]. Quantification of inter-frequency phase relationships, alongside frequency domain analysis, is thought to capture additional information relevant to the identification of non-linear signals. There was no significant change in either group in the SFS-C between cease of inhalational agent and eyes open. This suggests that analysis of phase relationships may not provide significant useful information regarding patient conscious state during the emergence phase.

The spectral edge frequency 95 (SEF95) is not believed to contribute to the BIS index but has been shown to correlate well with BIS values [5]. The SEF95 is displayed by the SEDline monitor (Masimo Corporation, Irvine, USA) although the manufacturer does not state if it forms part of the SEDline’s Patient State Index [29]. We found that the SEF95 increased from cease inhalational to eye opening for both groups, although the absolute value was significantly lower in the xenon group for most of the emergence period.

The Composite Cortical State (CCS) is an EEG derived measure of patient conscious state which is based on a neurophysiological model of the cortex. Its calculation depends on autoregressive moving average modelling as opposed to the Fourier transform that underlies the frequency domain analysis of the BIS and many other pEEG monitors [7]. The unscaled CCS index increased in both groups and the absolute value was similar between groups for most of the emergence period. A scaled version of the CCS is displayed by the Brain Anaesthesia Response Monitor (BAR) (Cortical Dynamics, Perth, Australia) as a measure of hypnotic depth and median BAR values for the participants are available in the Supplementary Digital Content.

Whilst the RBR increased over the emergence period for the sevoflurane group, it remained unchanged in the xenon group. This limits its usefulness as a predictor of responsiveness during xenon anesthesia. In contrast, the SEF95 and CCS increased significantly in both groups during the emergence period, despite the different molecular targets of these two agents. These parameters might have utility in the monitoring of xenon anesthesia and could potentially be useful measures of anesthetic depth for a broader range of agents, both predominantly GABAergic and non-GABAergic.

A delay between EEG collection and BIS index display has been proposed by previous authors as a potential source of error during relatively rapid xenon emergence [18, 20, 21]. Whilst the manufacturers of the BIS do not provide a definitive value for the time between EEG collection and changes in BIS index, an early publication describing the BIS suggested that calculation of the bispectrum requires averaging over 60 s [3]. There was no significant change in either the RBR or SFS in the xenon group during emergence. This would suggest that even with a reduction in processing ‘delay’, neither of these sub parameters are sensitive to changes in the spectral features of the EEG during xenon emergence.

The primary limitation of this study is that the number of participants was not determined by a power calculation designed to test the primary and secondary outcomes. Whilst a significant difference in BIS index values between anesthetic groups was identified, a failure to identify a difference between EEG parameter values between groups, or between the beginning and end of emergence, could reflect a type II error. The results of the study should be considered hypothesis generating rather than definitive.

The variability in response of pEEG monitors during the use of non-GABAergic anesthetic agents remains a significant challenge. We have identified that the relative beta ratio may not be a reliable indicator of emergence from the non-GABAergic agent xenon and identified two alternative EEG derived parameters, spectral edge frequency 95 and the composite cortical state, that might better reflect the transition to responsiveness. Future studies could address if these parameters are useful in identifying the transition to responsiveness during the use of other non-GABAergic agents and if incorporation of these parameters might improve the performance of pEEG monitors during non-GABAergic anesthesia.

## Supplementary Information

Below is the link to the electronic supplementary material.

Supplementary material 1 (DOCX 67.1 kb)
