# Supplementary material for: Performance of the bispectral index and electroencephalograph derived parameters of anesthetic depth during emergence from xenon and sevoflurane anesthesia
Source: J Clin Monit Comput. 2022 Apr 19;37(1):71–81. doi: 10.1007/s10877-022-00860-y (PMC9852153; doi:10.1007/s10877-022-00860-y)
Supplement: Supplementary file 1 — Supplementary material 1 (DOCX 67.1 kb) [file 10877_2022_860_MOESM1_ESM.docx]

Supplementary Digital Content

Brain Anesthesia Response Monitor (BAR) index values


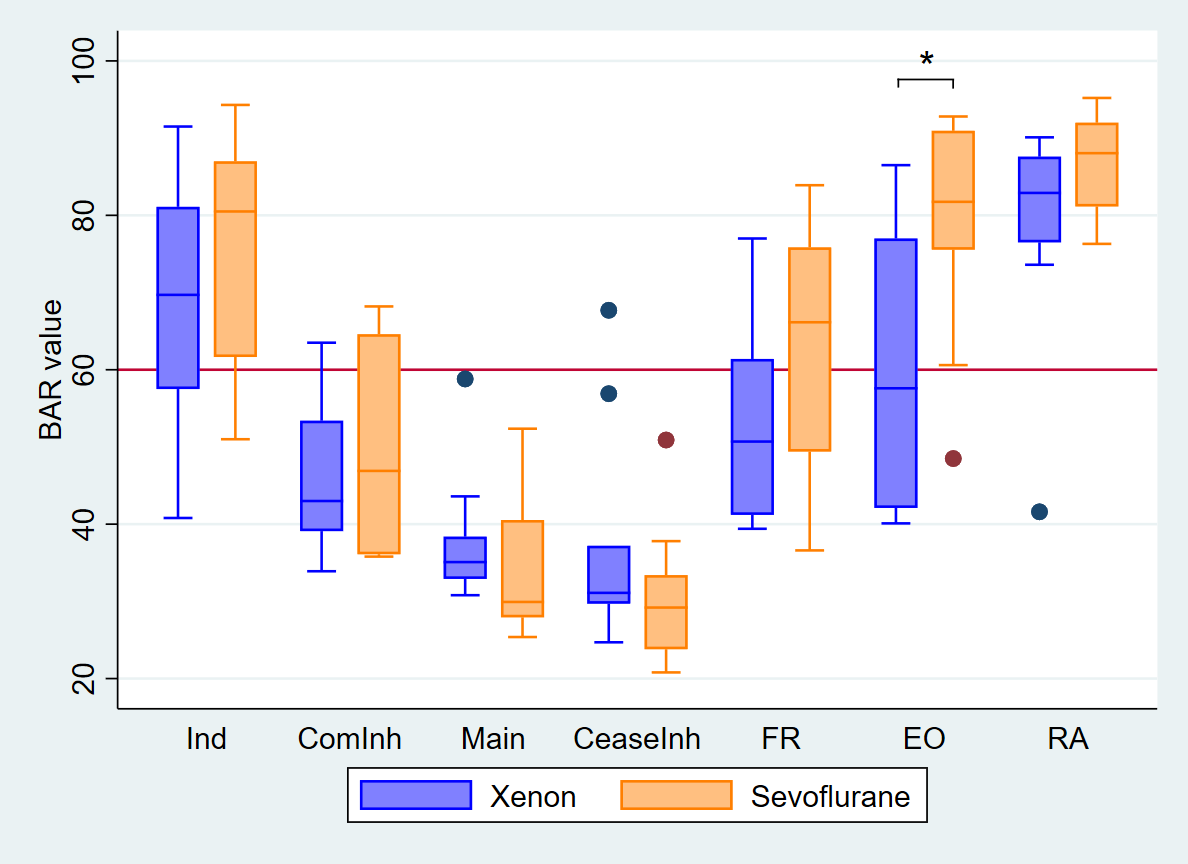


Comparison of BAR index values between xenon (blue) and sevoflurane (orange) groups during anesthesia and emergence. Median represented by line within box and the bounds of the boxes represent the 25^th^ and 75^th^ centiles. The whiskers represent values within 1.5x the interquartile range (IQR) and outside values (represented by dots) are values beyond this. Statistically significant differences (Wilcoxon rank sum) are indicated by an asterisk (* *P* = 0.03). Ind = induction, ComInh = commence inhalational, Main = maintenance, CeaseInh = cease inhalational, FR = first response, EO = eyes open, RA = removal of airway, BAR = Brain Anesthesia Response monitor.

Data acquired with BAR monitor (software version 2.8, firmware version 1.1.39, no artifact rejection). Xenon (Ind) median 81.1, IQR = [71.7 85.1], whiskers = [63.8 87.6]; Sevoflurane (Ind) median 80.7, IQR = [77.7 81.9], whiskers = [64.6 92.1], calculated offline using artifact rejection that is incorporated in updated BAR software (3.0) and firmware (2.0) revisions.
